# Investigation of In Vitro α‐Amylase Inhibitory, Antioxidant, Phytochemical Profiling, Molecular Docking, and Swiss ADME Analysis of *Magnolia zotictla*

**DOI:** 10.1002/cbdv.71677

**Published:** 2026-09-15

**Authors:** Muhammad Zahoor, Usra Shah, Marwa Bibi, Adnan Khan, Haseena, Amal Alotaibi

**Affiliations:** ^1^ Department of Biochemistry University of Malakand Chakdara Pakistan; ^2^ Department of Chemistry University of Malakand Chakdara Pakistan; ^3^ Department of Basic Science College of Medicine Princess Nourah bint Abdulrahman University Riyadh Saudi Arabia

**Keywords:** docking, enzyme inhibition, extract, GC–MS, HPLC, SwissADME

## Abstract

The current study was carried out to determine the in vitro antioxidant and antidiabetic potential and phytochemical profiling of crude extract obtained from the cone of *Magnolia zotictla*, and then to integrate it with computational chemistry. Phytochemical profiling was determined via HPLC and GC–MS analysis. In HPLC analysis, five compounds were identified, while five compounds were identified through GC–MS. The plant extract exhibited good antioxidant efficacy in the DPPH assay, highlighting its capacity to neutralize free radicals. The extract has an *IC*_50_ value of 123.8 µg/mL, while that of standard ascorbic acid was found to be 62.9 µg/mL. Similarly, the cone extract effectively inhibited α‐amylase and produced result, having *IC_50_* value of 58.17, while that of standard was 52.00. The docking results displayed interactions of the ligands with different receptors. In SwissADME analysis, many of the results were in accordance with drug discovery and development rules. The results reflect that the cones have potential antioxidant and antidiabetic effects. Comparing the *IC*_50_ values for both antioxidant and antidiabetic activity, it can be seen that the extracts have produced good results in antioxidant activity, which may be attributed to phenolic compounds present in the extract.

## Graphical abstract

Phytochemical profiling was determined via HPLC and GC–MS analysis. In HPLC analysis, five compounds were identified, while five compounds were identified through GC–MS. The plant extract exhibited good antioxidant efficacy in the DPPH assay, highlighting its capacity to neutralize free radicals. The extract has *IC*_5_₀ value of 123.8 µg/mL, while that of standard ascorbic acid was found to be 62.9 µg/mL. Similarly, the cone extract effectively inhibited *α*‐amylase and produced a result, having *IC*_5_₀ value of 58.17, while that of the standard was 52.00.

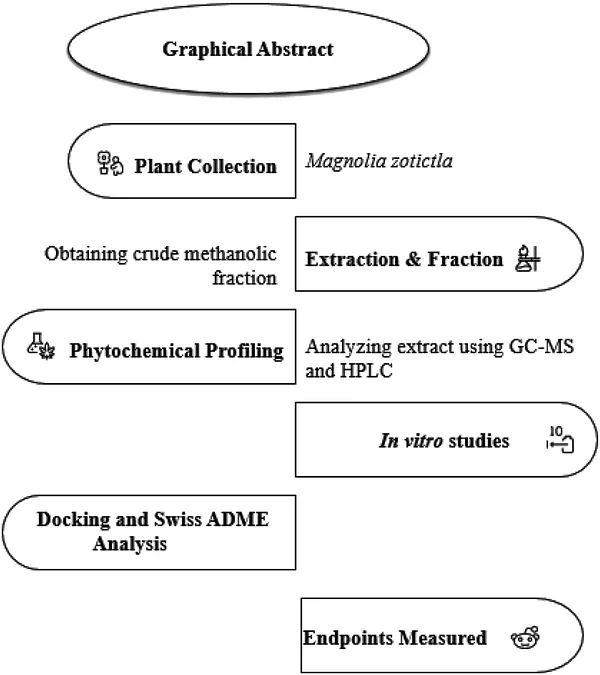

## 1 Introduction

For centuries, human being is using medicinal plants as a primary source of healthcare and treatment of various ailments [1]. Their therapeutic properties have been documented in traditional medicine systems in many cultures. Recently, interest is growing in exploring herbal and medicinal plants as a complementary or alternative approach to modern pharmaceuticals [2]. Medicinal plants have served healthcare systems throughout history. Throughout history, medicinal plants have played a crucial role in healthcare practices. The early civilizations, including Greeks, Egyptians, Romans, and Chinese have used plant‐derived herbal remedies as a source of health care and prevention of various diseases [3]. Plant‐derived health care remedies are getting recognitions as they possess therapeutic potential [4]. They are being a source of bioactive constituents, including phenolics, alkaloids, terpenoids, and flavonoids, due to which plant extracts have a wide array of pharmacological efficacy [5, 6]. Medicinal plants are used in health care as they are effective in terms of therapeutic potential, low cost, and being least toxic than synthetic medications. Access to medicinal plants in many parts of the world, especially developing countries, signifies plant‐based medication, as conventional medication in these areas is limited [7]. Scientific studies support medicinal potential of plants by providing evidences on bioactive constituents of plants, their mechanistic studies, and their safety profile [8]. Various spectrometric, chromatographic techniques, and enzymatic assays are used to characterize, quantify, and assess the medicinal efficacy of the metabolites present in plants [9, 10]. The secondary metabolites present in plants are considered as essential phytochemicals and include flavonoids, terpenoids, and phenolic constituents. They are not involved in growth and development of plants but often have important ecological roles like providing defense from herbivores and pathogens [11]. Methods such as nuclear magnetic resonance (NMR) spectroscopy and liquid chromatography‐mass spectrometry (LC‐MS) are utilized to identify and quantify secondary metabolites in plant extracts [12].

*Diabetes mellitus* (DM) is a chronic metabolic disorder characterized by higher glucose levels in blood and is usually associated with low secretion of insulin as well as insulin resistance and lipid profile [13, 14]. Currently, DM is a global health concern and is providing challenges to current therapeutics [15, 16]. Diabetes, if left untreated, will cause severe complications, including nonalcoholic fatty liver disease, heart failure, renal failure, and vascular disturbances [17]. In the management of diabetes, new drugs are still the demands of the health care system. Many plants are known to have promising antidiabetic potential [18].

*Magnolia* genus comprises of more than 300 species that are known for their pharmacological, medicinal, and ornamental significance [19]. Phytochemical analysis of *Magnolia* has revealed the presence of bioactive components, including phenolic acids, flavonoids, tannins, alkaloids, and terpenoids. These compounds have demonstrated strong antidiabetic, anti‐inflammatory, and antioxidant efficacy [20]. Extracts from several species of *Magnolia* have exhibited potent radical scavenging efficacy and inhibitory potential against *α*‐glucosidase and *α*‐amylase and suggest their significance as a therapeutic source for diabetes and related disorders [21]. Literature has reported studies on leaves and bark sections, while the cone section has remained largely unexplored. Besides this, studies on cone section of *Magnolia grandiflora* have been reported [22], but no direct study has been reported on phytochemical investigation of the cone section of *Magnolia zotictla*.

The current study aimed to integrate the in vitro antidiabetic and antioxidant effects of *M. zotictla cones* extract with the phytochemicals present in the extract and then to validate the findings by computational approaches, including molecular docking and SwissADME analysis. To the best of our knowledge, no such study has been reported previously, and this will be the first study to integrate in vitro *effects*, phytochemical profile, and computational studies on *M. zotictla*.

## 2 Experimental Section

### 2.1 Chemical Reagents

Research‐grade chemicals and solvents were used in this study and were purchased from a local chemical supplier and used as such without further purification. *α*‐Amylase (CAS no 9000‐90‐2), DNSA (CAS no 609‐99‐4), soluble starch (CAS no 9005‐84‐9), sodium phosphate (CAS no 7558‐80‐7), acarbose (CAS no 56180‐94‐0), methanol (CAS no 67‐56‐1), and ethanol (CAS no 64‐17‐5).

### 2.2 Plant Collection and Authentication

The cones of *M. zotictla* plant were collected from local area of Chakdara, Dir Lower, KPK, Pakistan. *Magnolia zotictla* was authenticated by taxonomist Gul Rahim, Department of Botany, University of Malakand. A voucher specimen (UOM / bot grd /346) was deposited in the Botanical Garden, University of Malakand.

### 2.3 Extraction

The cones of *M. zotictla* were rinsed with tap water to remove dirt. The cones were dried under shade and then ground to fine powder, and 400 g of dry powder was obtained, which was then macerated in methanol, and 3 times the volume of the solvent was added, and were stirred twice a day for about 2 weeks for effective extraction. After which, the solution was filtered and then concentrated by a rotary evaporator at 35°C–4°C, after which the extract was dried at room temperature, and a hard mass was obtained called crude or methanolic extract, which was stored in an airtight bottle and kept in a refrigerator at 4°C before further use.

### 2.4 Preliminary Phytochemical Analysis

#### 2.4.1 High‐Performance Liquid Chromatography (HPLC) Analysis

The phytochemical analysis was carried out using high‐performance liquid chromatography (HPLC) technique. The HPLC protocol was established for *M. zotictla* according to available standards. The extract solution was made in HPLC‐grade methanol and then subjected to HPLC system. All the necessary components of HPLC were installed with the system. Standard calibration curves were created with known concentrations of compound for effective separation. The extract was injected and then eluted, and compounds were identified from their retention time by cross‐matching with those of external standard run in their pure form. HPLC system model 1260 was purchased from Agilent manufacturer, USA. The system was connected to UV–vis detector operating at variable wavelengths (250–320 λ). C18 column was used with dimensions of (250 × 4.6 mm, 5 µm particle size) (S.no P.N. 990967‐902, Agilent manufacturer, USA) for separation of the two solvents consisting of water containing 0.1% (v/v) acetic acid and methanol. The elution was carried out using a gradient program. The column temperature was 30°C, and flow rate was 1.0 mL min^−^^1^ [23].

#### 2.4.2 Gas Chromatography–Mass Spectrometry (GC–MS) Analysis

To determine the photochemical analysis via GC–MS, crude extract of *M. zotictla* was subjected to GC–MS analysis. Gas chromatograph (model USB‐393752, Agilent manufacturer) was equipped with capillary column (having dimensions of 3 m × 0.25 mm diameter × 0.25 µm film thickness). The system was operating with an ionization energy of 70 eV in electron impact mode. Temperature was ramped to 180°C at a rate of 6°C per minute, and this temperature was kept for 5 min. The temperature was then ramped to 280°C and was maintained for 20 min. Injector temperature was 220°C, while that of detector was 290°C. *n*‐Pentane was used for dilution of samples in 1/1000 ratio. Flow rate of carrier gas (Helium) was 1 mL/min. The sample, after getting eluted, was ionized and then identified via mass spectrometer on the basis of *m/z* ratio. Compounds were identified by using retention time, correlation of spectral data with NIST and Wiley Library, as well as fragmentation pattern [24].

### 2.5 Antioxidant Activity

#### 2.5.1 DPPH Radical Scavenging Activity Assay

The DPPH assay was carried out according to previously reported protocols. DPPH solution (2 mL) was mixed with 2 mL solution of samples having concentrations of 1000, 500, 250, 125, and 62.5 µg/mL. The reaction mixture was kept in dark for 30 min, and absorbance was recorded at 517 nm against blank. Ascorbic acid was used as positive control [25].

### 2.6 Antidiabetic Potential of Extract

#### 2.6.1 *α*‐Amylase Inhibition Assay

*α*‐Amylase inhibition assay was carried out as per previous protocols. Various concentrations were made in 96‐well plates, to which 50 µL phosphate buffer (pH = 6.8, conc = 100 mM) was added, followed by the addition of *α*‐amylase (10 µL, 2 U/mL). The reaction mixture was incubated at 37°C for 20 min. After which, 1% starch (10 µL) was added and then incubated for 30 min, followed by addition of DNS (100 µL) and boiled at 100°C for 5 min, absorbance was taken at 656 nm, and percentage inhibition and *IC*_50_ were calculated [26]. The following formula was used for determination of percentage inhibition.

Percentageinhibition=(1−S/C)

where *S* is reading of absorbance in the presence of test sample, while *C* is the reading of absorbance without test sample (control).

### 2.7 Molecular Docking

The in silico studies (molecular docking) were carried out using MOE software. Proteins were downloaded from PDB. Compounds were bind with enzyme, and minimum energy structures were obtained. After docking, π–π interactions and hydrogen bonding were analyzed. The compounds identified in HPLC and GC–MS were docked against *α*‐amylase [27].

### 2.8 SwissADME analysis

Properties like pharmacokinetic profile, drug likeness, and medicinal chemistry of the compounds identified by HPLC and GC–MS were screened by SwissADME. Structure of compounds were drawn on Chem Draw and then imported into SwissADME using SMILES, and data were generated by system [28, 29, 30, 31]. SwissADME is accessible at http://www.swissadme.ch.

## 3 Results

### 3.1 Phytochemical Analysis

The phytochemical composition of *M. zotictla* crude extract was evaluated using HPLC and GC–MS analysis. A number of compounds were present in crude extract, out of which a few were identified as described below.

#### 3.1.1 HPLC Analysis

**FIGURE 1:**
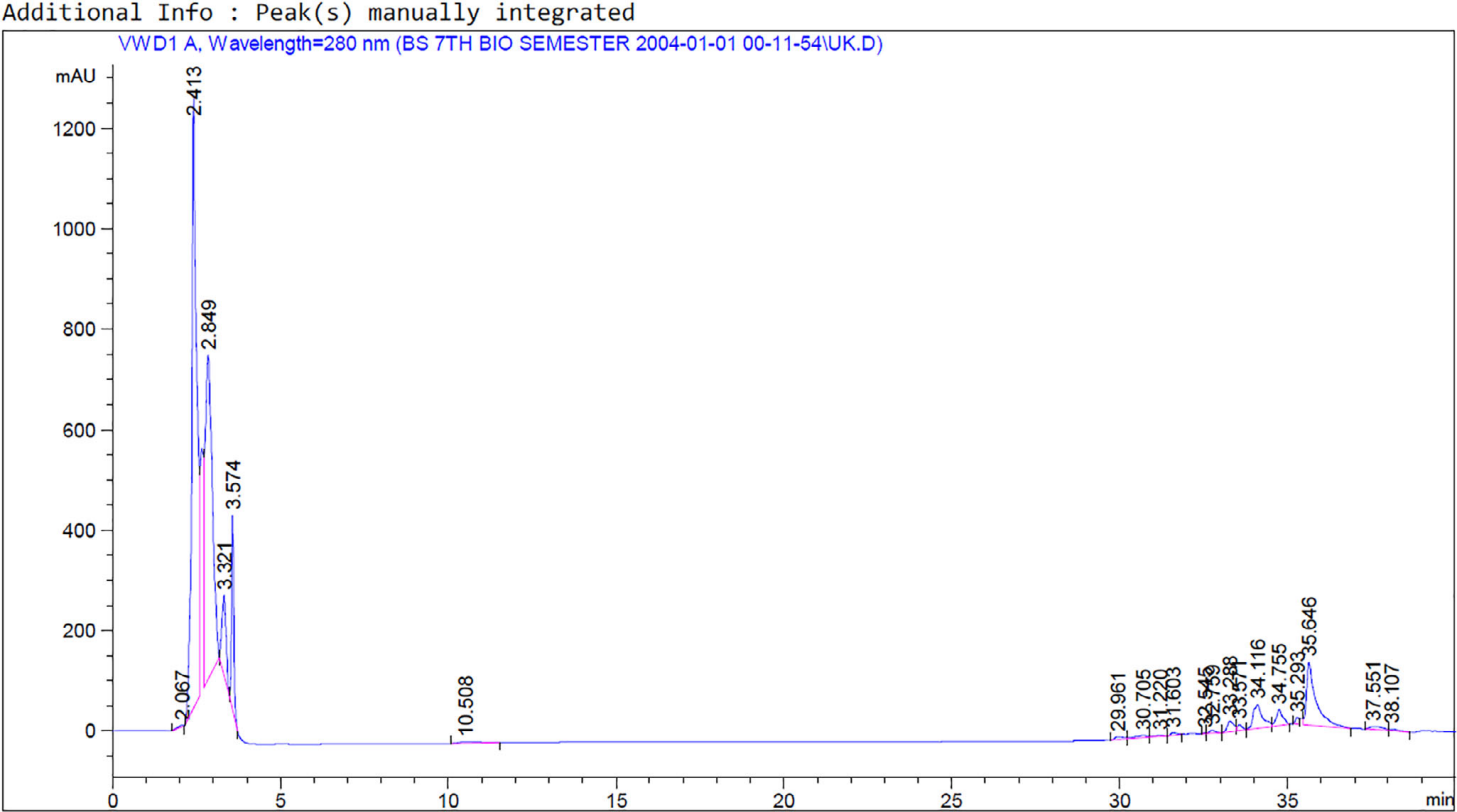
HPLC chromatogram of *M. zotictla* cone extract.

The HPLC chromatogram of crude extract shows many peaks, out of which a few were identified as per comparison with standards and reported data. Table 1 shows the phytochemicals identified by HPLC upon comparing to standard, while HPLC chromatogram is shown in Figure 1.

**TABLE 1:** HPLC profile of crude extract of cones of *M. zotictla*.

S. No	Compound name	Peak area (mAU*s)	Retention time (min)	Reference
1	Hydroxy benzoic acid	36.9172	2.413	Standard
2	Ascorbic acid	1.5712	10.508	Standard
3	Quercetin	77.87	32.759	Standard
4	Rutin	104.42	35.293	Standard
5	Catechin hydrate	2507.67407	5.55	Standard

#### 3.1.2 GC–MS Analysis

**FIGURE 2:**
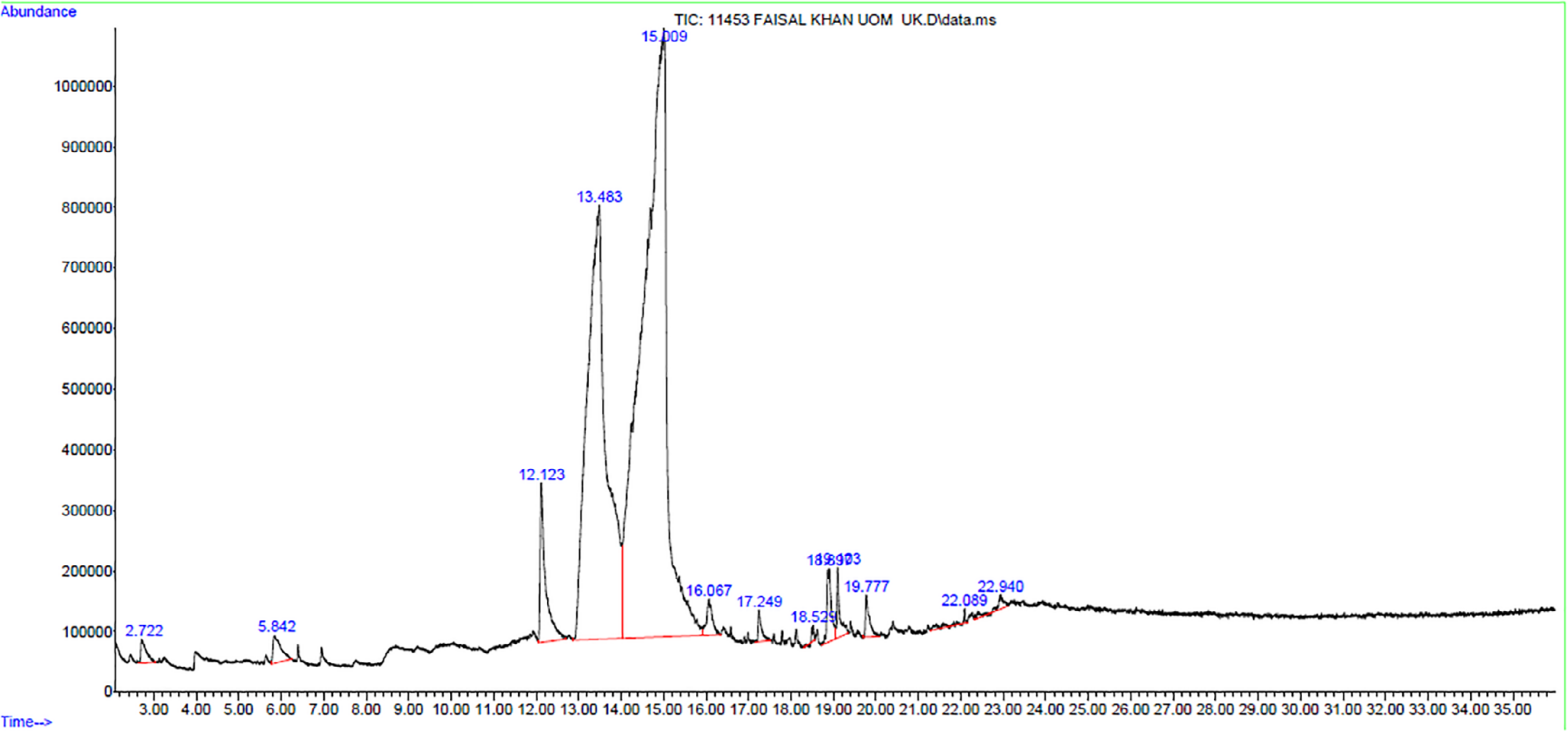
GC–MS chromatogram of crude extract of *M. zotictla* cone.

Table 2 presents the results of the GC–MS, and it was found that *M. zotictla* crude extract has known phytochemicals. The detected compounds are secondary metabolites of plant and have therapeutic potential, along with innate defensive functions performed by them. Table 2 summarizes secondary metabolites of identified in the extract. The GC–MS chromatogram is shown in Figure 2.

**TABLE 2:** GC–MS profile of crude extract of *M. zotictla* cone.

S. No	Compound	Peak area	Retention time
1	Butanoic acid	227,268,678	2.480
2	Tridecanoic acid	5,510,547	16.067
3	*n‐*Hexadecanoic acid	2,858,549	17.249
4	6‐Octadecanoic acid	763,923	18.529
5	Oleic acid	8,803,408	

### 3.2 Antioxidant Activity

#### 3.2.1 DPPH Radical Scavenging Assay

The antioxidant activity was carried out by DPPH assay as per previously reported protocols. Ascorbic acid was used as standard. Table 3 presents the results of DPPH assay. The *IC*_50_ value of extract was found to be 123.86 µg/mL, while that of standard ascorbic acid was found to be 62.9 86 µg/mL

**TABLE 3:** DPPH inhibition assay results of selected plant cone extract.

Sample	Concentration	% Inhibition	*IC*_50_ (µg/mL)
Extract	62.5	48.23	123.86
	125	50.46	
	250	55.29	
	500	66.17	
	1000	77.19	
Ascorbic acid	62.5	48.09	62.9
	125	52.01	
	250	62.01	
	500	73.76	
	1000	95.56	

#### 3.2.2 Antidiabetic Activity

##### 3.2.2.1 *α*‐Amylase Activity

The enzyme inhibition procedure employed while studying α‐amylase was according to the reported literature method [26]. Acarbose was used as a standard drug in *α*‐amylase inhibition assay. The results are summarized in Table 4. The standard acarbose has *IC*_50_ of 52 µg/mL, whereas the extract has its value as 58.17 µg/mL, showing that extract contains some metabolites to cure diabetes; however, if isolated in pure form, the efficacy would have been high.

**TABLE 4:** Inhibition of *α*‐amylase by cone extract.

Sample	Concentration	% Inhibition	*IC*_50_ (µg/mL)
Extract	62.5	53.72	58.17
	125	62.72	
	250	66.97	
	500	72.19	
	1000	76.96	
Acarbose	62.5	48.09	52.00
	125	52.01	
	250	62.01	
	500	73.76	
	1000	80.30	

### 3.3 In Silico Studies

#### 3.3.1 Docking Studies With α‐Amylase

**FIGURE 3:**
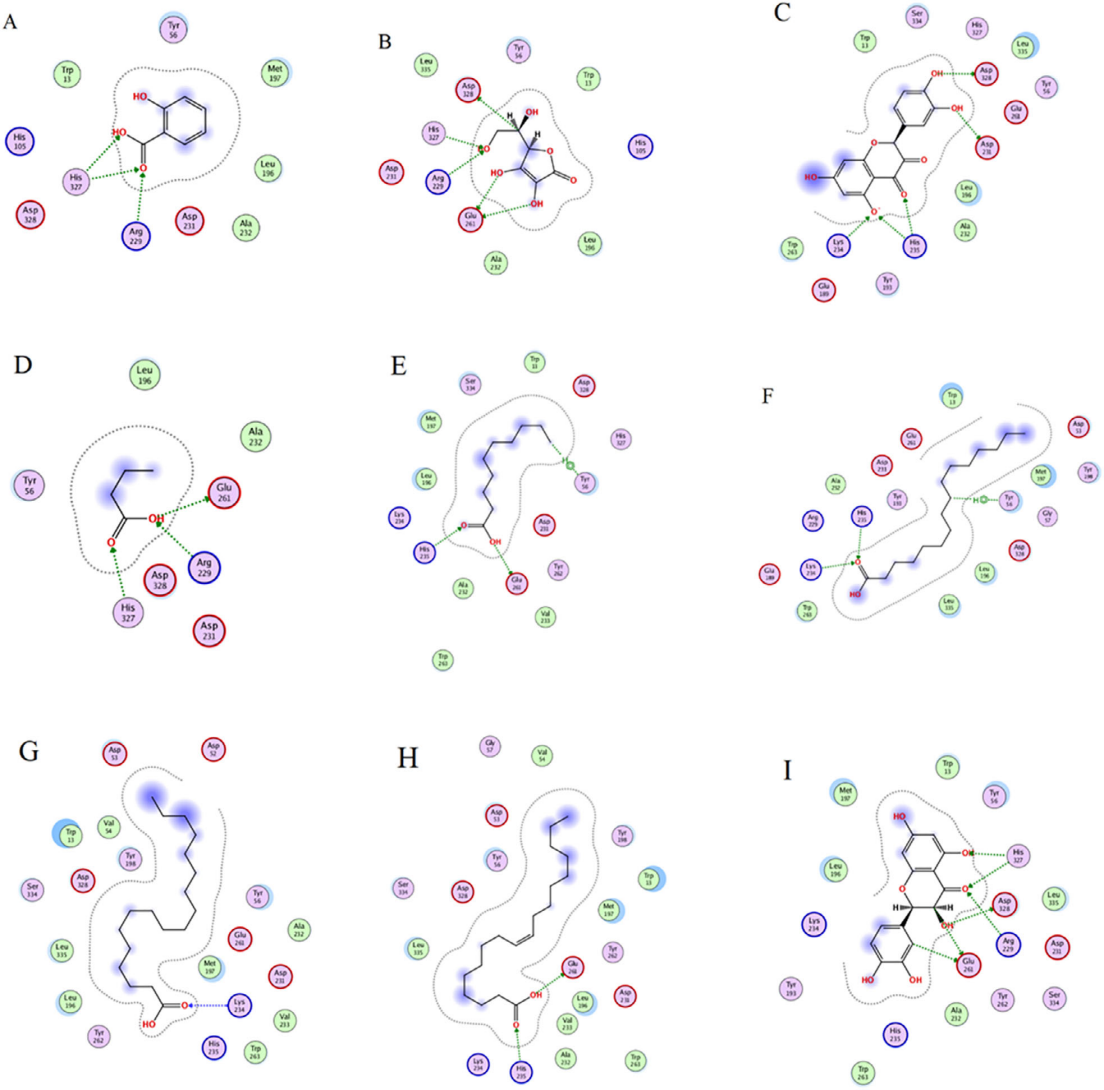
Molecular docking of compounds with amylase enzyme: (A) Hydroxy benzoic acid, (B) ascorbic acid, (C) quercetin, (D) butanoic acid, (E) tri‐decanoic acid, (F) *n*‐hexadecanoic acid, (G) octadecanoic acid, (H) oleic acid, and (I) catechin.

Figure 3A represents docking of hydroxy benzoic acid with α‐amylase, which formed three covalent bonds: two with HIS327 and one with ARG229, with distances of 2.91, 3.09, and 3.46 Å and energies of −0.5, −3.2, and ‐1.4 kcal/mol, respectively. Binding affinity was 4.3. Figure 3B shows docking of ascorbic acid with *α*‐amylase, which formed five covalent bonds two with GLU261 and one each with ASP328, ARG229, and HIS327 with distances of 2.95, 3.02, 3.33, 3.09, and 3.13 Å with energies of −3.7, −3.8, −0.9, −2.5, and −0.9 kcal/mol, respectively, and docking score was 4.3. Figure 3C illustrates docking of quercetin with *α*‐amylase, which formed five covalent bonds two with HIS235 and one each with ASP328, ASP231, and LYS234 with distances of 3.03, 3.00, 2.97, 2.93, and 2.87 Å and energies of −0.7, −1.4, −3.8, −2.4, and −8.8 kcal/mol, respectively, and docking affinity was 6.0. Figure 3D shows docking of butanoic acid with *α*‐amylase, which formed three covalent bonds, one each with GLU261, ARG229, and HIS327, with distances of 3.23, 2.97, and 3.09 Å, respectively, and energies of −0.5, −1.6, and −2.4 kcal/mol, respectively. Binding score was 3.6. Figure 3E represents the docking of tri‐decanoic acid with α‐amylase, forming one hydrogen pi bond and two covalent bonds. The pi bond was between aromatic ring and TYR56 having distance of 3.61 Å and energy of −0.6 kcal/mol, and covalent bonds were formed with GLU261 and HIS235 having distances of 2.94 and 2.92 Å and energies of −3.2 and −2.3 kcal/mol. Binding affinity was 4.9. Figure 3F illustrates docking of *n*‐hexadecanoic acid with α‐amylase, which formed one hydrogen pi bond and two covalent bonds. The pi bond was between aromatic ring and TYR56, having distance of 4.43 Å and energy of −0.5 kcal/mol, and covalent bonds were formed with LYS234 and HIS235, having distances of 3.36 and 2.92 Å and energies of −1.2 and −1.6 kcal/mol, respectively, with docking score of 6.2. Figure 3G shows docking of octadecanoic acid with *α*‐amylase; they establish a covalent bond with LYS234 with a distance of 3.21 Å and energy of −1.4 kcal/mol, with docking score of 6.7. Figure 3H indicates docking of oleic Acid with *α*‐amylase; they formed three bonds, two with GLU261 and one with HIS235, with distances of 2.92, 2.96, and 2.82 Å, and energies of −2.0, −1.0, and −3.0 kcal/mol. Docking score was 6.4. Figure 3I indicates docking of catechin and α‐amylase, which formed six bonds: two each with GLU 261 and HIS327, and one each with ASP328 and AGR229 with distances of 3.38, 3.39, 3.01, 3.35, 3.02, and 3.33 Å and energies of −0.7, −0.5, −0.7, −0.9, −0.8, and −2.4 kcal/mol. Docking score was 6.1. The results are tabulated in Table 5.

**TABLE 5:** Docking studies of phytochemicals identified with *α*‐amylase.

Ligand	Bond	Residue	Distance (Å)	Energy (kcal/mol)	Binding affinity
Hydroxy benzoic acid	Three covalent bonds	2 with HIS327, one with ARG229	2.91, 3.09, 3.46	−0.5, −3.2, −1.4	4.3
Ascorbic acid	Five covalent bonds	Two with GLU261, one each with ASP328, ARG229 and HIS327	2.95, 3.02, 3.33, 3.09, 3.13	−3.7, −3.8, −0.9, −2.5, −0.9	4.3
Quercetin	Five covalent bonds	Two with HIS235, one each with ASP328, ASP231 and LYS234	3.03, 3.00, 2.97, 2.93, 2.87	−0.7, −1.4, −3.8, −2.4, −8.8	6.0
Butanoic acid	Three covalent bonds	One each with GLU261, ARG229 and HIS327	3.23, 2.97, and 3.09	−0.5, −1.6, −2.4	3.6
tri‐decanoic acid	One H‐pi and two covalent bonds	TYR56, one covalent bond each with GLU261 and HIS235	3.61, 2.94, 2.92	−0.6, −3.2, −2.3	4.9
*n*‐Hexadecanoic acid	One H‐pi and two covalent bonds	TYR56, one covalent bond each with LYS234, HIS235	4.43 Å, 3.36 2.92	−0.5, −1.2 −1.6	6.2
Octadecanoic acid	One covalent bond	LYS234	3.21	−1.4	6.4
Oleic Acid	Three covalent bonds	Two with GLU261 and one with HIS235	2.92, 2.96, and 2.82	−2.0, −1.0, −3.0	6.4
Catechin	Six covalent bonds	Two with each GLU261 and HIS327 and one each with ASP328 and AGR229	3.38, 3.39, 3.01, 3.35, 3.02, 3.33	−0.7, −0.5 0.7, −0.9, −0.8. −2.4	6.1

#### 3.3.2 SwissADME Analysis of the Phytochemicals Identified by HPLC and GC–MS

##### 3.3.2.1 Analysis of Physicochemical Properties

The physicochemical properties of the identified phytochemicals are tabulated in Table 6. Molecular weight of hydroxy benzoic acid was 138.12, number of hydrogen bond donors were 2, topological polar surface area was 57.53, log *P* was found to be 1.24 and hydrogen bond acceptors were 3, number of rotatable bond was 1, while the molar refractivity was found to be 35.42. These parameters for ascorbic acid were found to be 176.12, 4, 107.22, −1.24, 6, 2, and 35.12. The results for quercetin were determined to be 302.24, 5, 131.36, 1.23, 7, 1, and 78.04. For catechin, the results were 322.27, 6, 136.68, 0.36, 8, 1, and 77.80. Similarly, for butanoic acid, the results were 88.11, 1, 37.30, 0.68, 2, 2, and 23.1. For *n*‐decanoic acid and *n*‐hexadecanoic acid, the results were 172.26, 1, 37.30, 3.00, 2, 8, and 51.6, and 256.42, 1, 37.30, 5.20, 2, 14, and 80.80, respectively. Octadecanoic acid has molecular weight of 284.48, H‐bond donors were 1, topological polar surface area was 37.30, log *P* was 5.93, hydrogen bond acceptors were 2, number of rotatable bonds were 16, and molar refractivity was 90.41. These parameters for oleic acid were found to be 282.46, 1, 37.30, 5.65, 2, 15, and 89.94.

**TABLE 6:** Physicochemical properties of phytochemical determined by SwissADME database.

S.No	Ligands name	M.W (g/mol)	M. Formula	nAHA	Nha	F.Csp [3]	nRB	nHBD	nHBA	TPSA (Å [2])	MR
1	Hydroxy benzoic acid	138.12	C_7_H_6_O_3_	6	10	0.00	1	2	3	57.53	35.42
2	Ascorbic acid	176.12	C_6_H_8_O_6_	0	12	0.50	2	4	6	107.22	35.12
3	Quercetin	302.24	C_15_H_10_O_7_	16	22	0.00	1	5	7	131.36	78.04
4	Catechin	322.27	C_15_H_14_O_8_	12	23	0.13	1	6	8	136.68	77.80
5	Butanoic acid	88.11	C_4_H_8_O_2_	0	6	0.75	2	1	2	37.30	23.11
6	Tri‐decanoic acid	172.26	C_10_H_20_O_2_	0	12	0.90	8	1	2	37.30	51.96
7	*n*‐Hexadecanoic acid	256.42	C_16_H_32_O_2_	0	18	0.94	14	1	2	37.30	80.80
8	Octadecanoic acid	284.48	C_18_H_36_O_2_	0	20	0.94	16	1	2	37.30	90.41
9	Oleic acid	282.46	C_18_H_34_O_2_	0	20	0.83	15	1	2	37.30	89.94

*Abbreviations*: TPSA = topological polar surface area, MR = molar refractivity, nHBD = No. H‐bond donors, nHBA = No. H‐bond acceptors, nRB = No. rotatable bonds, FCsp [3] = fraction of SP^3^ carbon, nAHA = No. arom. heavy atom, nHA = No. heavy atom, M.W = molecular weight, M.Formula = molecular formula.

##### 3.3.2.2 Lipophilicity and Water Solubility

Table 7 displays lipophilicity and solubility in water of the phytochemicals determined in the plant. The log *P*_o/w_ for hydroxy benzoic acid was 1.24, indicating its moderate lipophilicity. Log *S* indicates the solubility of a compound in water, which was found to be −2.50 for hydroxy benzoic acid, indicating that its solubility in water is good. Log *P*_o/w_ for ascorbic acid is −1.24 and is poorly lipophilic, while its log *S* value signifies its solubility in water and was found to be 0.23. The log *P*_o/w_ for quercetin and catechin were 1.23 and 0.36, while their log *S* values were −3.16 and −2.46. The log *P*_o/w_ value for butanoic acid was found to be 0.68, indicating its modest lipophilicity, and its log *S* value was −0.75.

**TABLE 7:** Water solubility and lipophilicity of phytochemical determined by SwissADME.

S.NO	Ligands name	Log *S* (ESOL)	Consensus log *P*_o/w_	Solubility class	Log *S* (Ali)	Solubility class	Log *S* (SILICOS‐IT)	Solubility class
1	Ascorbic acid	0.23	−1.24	Highly soluble	−0.10	Very soluble	1.49	Soluble
2	Hydroxy benzoic acid	−2.50	1.24	Soluble	−3.10	Soluble	−1.17	Soluble
3	Catechin	−2.46	0.36	Soluble	−2.92	Soluble	−2.03	Soluble
4	Quercetin	−3.16	1.23	Soluble	−3.91	Soluble	−3.24	Soluble
5	Tri‐decanoic acid	−2.96	3.00	Soluble	−4.58	Moderately soluble	−2.87	Soluble
6	Octadecanoic acid	−5.73	5.93	Moderately soluble	−8.87	Poorly soluble	−6.11	Poorly soluble
7	Oleic acid	−5.41	5.65	Moderately soluble	−8.26	Poorly soluble	−5.39	Moderately soluble
8	Butanoic acid	−0.75	0.68	Very soluble	−1.15	Very soluble	−0.35	Soluble
9	*n‐*Hexadecanoic acid	−5.02	5.20	Moderately soluble	−7.77	Poorly soluble	−5.31	Moderately soluble

Similarly, log *P*_o/w_ for tri‐decanoic acid, octadecanoic acid, *n*‐hexadecanoic acid, and oleic acid was 3.00, 5.93, 5.20, and 5.65, indicating their highest lipophilicity, while their log *S* values were −2.96, −5.02, −5.73, −5.41, and −4.07, indicating their poor solubility in water.

##### 3.3.2.3 Pharmacokinetic Profile

**FIGURE 4:**
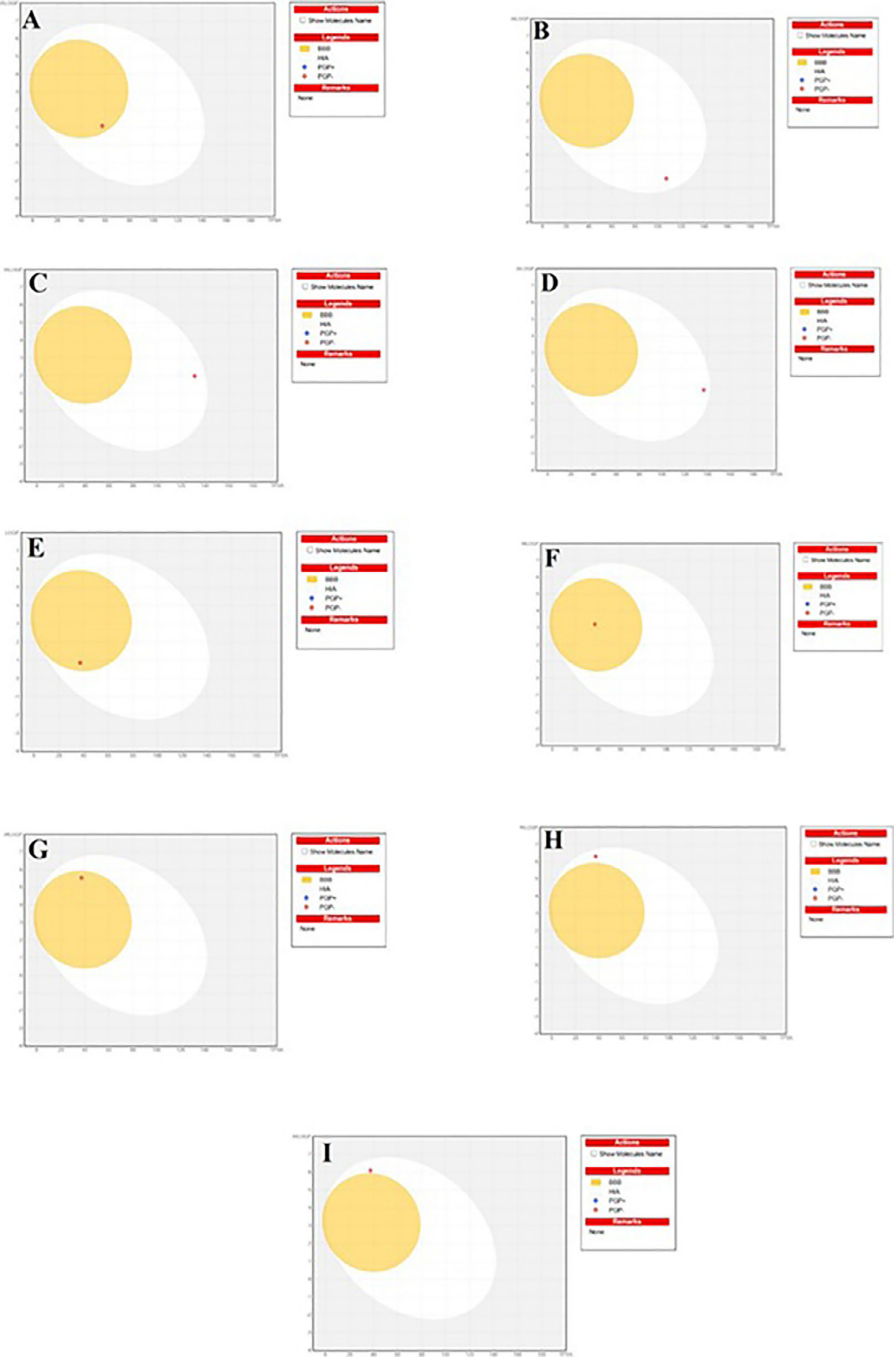
Boiled graph representations of compounds. (A) hydroxy benzoic acid, (B) ascorbic acid, (C) quercetin, (D) butanoic acid, (E) tri‐decanoic acid, (F) *n*‐hexadecanoic acid, (G) octadecanoic acid, (H) oleic acid, and (I) catechin.

As per SwissADME database, the gastrointestinal absorption of the compounds was found to be high, therefore, these compounds can be orally administered. Hydroxy benzoic acid is graphically depicted in Figure 4A, the compound has ability to cross the blood–brain barrier (BBB), and this compound does not act as a substrate of *P‐*glycoprotein. Hydroxy benzoic acid has no action against CYP1A2, CYP2C19, CYP2C9, CYP3A4, and CYP2D6. Figure 4B represents the boiled egg graph of ascorbic acid; the compound has no ability to permeate BBB. This compound does not act as a substrate of *P*‐glycoprotein. Ascorbic acid has no action against CYP1A2, CYP2C19, CYP2C9, CYP3A4, and CYP2D6. Quercetin is graphically depicted in Figure 4C, the compound has ability to cross the BBB, and this compound does not act as a substrate of *P‐*glycoprotein. Butanoic acid has no action against CYP2C19 and CYP2C9. Butanoic acid is graphically depicted in Figure 4D, the compound has ability to cross the BBB, and this compound does not act as a substrate of *P‐*glycoprotein. The compound has no action against CYP1A2, CYP2C19, CYP2C9, CYP3A4, and CYP2D6. Tri‐decanoic acid has no action against CYP2C19 and CYP2C9. Butanoic acid is graphically depicted in Figure 4E, the compound has ability to cross the BBB, and this compound does not act as a substrate of *P‐*glycoprotein. The compound has no action against CYP1A2, CYP2C19, CYP2C9, CYP3A4, and CYP2D6. *n*‐Hexadecanoic acid is graphically depicted in Figure 4F, the compound has ability to cross the BBB, and this compound does not act as a substrate of *P‐*glycoprotein. The compound has no action against CYP3A4, CYP2C19, and CYP2D6. The compound can inhibit CYP1A2 and CYP2C9. Octadecanoic acid is graphically depicted in Figure 4G, the compound has ability to cross the BBB, and this compound does not act as a substrate of *P‐*glycoprotein. The compound has no action against CYP2C19, CYP2C9, CYP3A4, and CYP2D6 and can inhibit CYP1A2. Oleic acid is graphically depicted in Figure 4H, the compound has ability to cross the BBB, and this compound does not act as a substrate of *P‐*glycoprotein. The compound has no action against CYP2C19, CYP2C9, CYP3A4, and CYP2D6 and can inhibit CYP1A2. The pharmacokinetics data of the compounds are summarized in Table 8.

**TABLE 8:** Pharmacokinetics properties of phytochemical determined by SwissADME database.

S. No.	Ligands name	GI absorption	BBB permeant	P‐gp substrate	CYP1A2 inhibitor	CYP2C19 inhibitor	CYP2D6 inhibitor	CYP2C9 inhibitor	CYP3A4 inhibitor	Log *K*_p_ (skin permeation)
1	Quercetin	High	No	No	Yes	No	Yes	No	Yes	−7.05 cm/s
	Hydroxy benzoic acid	High	yes	No	Yes	No	Yes	No	No	−5.54 cm/s
2	Ascorbic acid	High	No	No	No	No	No	No	No	−8.54 cm/s
3	Catechin	High	No	No	No	No	No	No	No	−7.93 cm/s
4	*n*‐Hexadecanoic acid	High	Yes	No	Yes	No	No	Yes	No	−2.77 cm/s
5	Tri‐decanoic acid	High	Yes	No	No	No	No	No	No	−4.45 cm/s
6	Butanoic acid	High	Yes	No	No	No	No	No	No	−6.28 cm/s
7	Oleic acid	High	No	No	Yes	No	No	Yes	No	−2.60 cm/s
8	Octadecanoic acid	High	No	No	Yes	No	No	No	No	−2.19 cm/s

##### 3.3.2.4 Drug Likeness

Table 9 shows the drug likeness of compounds determined by HPLC and GC–MS. Hydroxy benzoic acid, ascorbic acid, butanoic acid, and tri‐decanoic acid did not violate drug‐likeness parameters, including Veber, Lipinski, and Egan rules, but violated Muegge and Ghose rules; this means the drug is too small and cannot be optimize in powerful drug. Quercetin follows all the five parameters and rules of drug likeness. Catechin violates Lipinski and Muegge rules; it means the drug has more hydrogen bond donor therefore it maybe too polar. *n*‐Hexadecanoic acid, octadecanoic acid, and oleic acid did not violate some of the drug‐likeness parameters, including Veber and Ghose rules of drug‐likeness, but it violates Muegge, Lipinski, and Egan rules; it means the drug has too many rotatable bonds and has high lipophilicity. The bioavailability score of these compounds was 0.85 for hydroxy benzoic acid, butanoic acid, *n*‐hexadecanoic acid, tri‐decanoic acid, oleic acid, and octadecanoic acid, 0.55 for quercetin and catechin, while 0.56 for ascorbic acid.

**TABLE 9:** Drug likeness of the phytochemical determined by SwissADME database.

Drug likeness rules						
Ligands name	Muegge	Lipinski	Egan	Veber	Ghose	Bioavailability score
Ascorbic acid	Violation: MW<200	0 violation	Yes	Yes	1 violation WLOGP<‐0.4	0.56
Hydroxy benzoic acid	1 violation: MW<200	0 violation	Yes	Yes	2 violations MR<40, MW<160,	0.85
Catechin	1 violation: H‐don>5	1 violation NH&OH>5	1 violation: TPSA>131.6	Yes	Yes	0.55
Quercetin	Yes	0 violation	Yes	Yes	Yes	0.55
Tri‐decanoic acid	1 violation: MW<200	0 violation	Yes	Yes	Yes	0.85
Butanoic acid	2 violations: MW<200	0 violation	Yes	Yes	2 violations: MR<40, MW<160,	0.85
Octadecanoic acid	2 violations: XLOGP3>5, Rotors>15	1 violation: MLOGP>4.15	1 violation: WLOGP>5.88	1 violation: Rotors>10	1 violation: WLOGP>5.6	0.85
*n‐*Hexadecanoic acid	1 violation: XLOGP3>5	1 violation: MLOGP>4.15	Yes	1 violation: Rotors>10	Yes	0.85
Oleic Acid	1 violation: XLOGP3>5	1 violation: MLOGP>4.15	1 violation: WLOGP>5.88	1 violation: Rotors>10	1 violation: WLOGP>5.6	0.85

##### 3.3.2.5 Medicinal Chemistry

**FIGURE 5:**
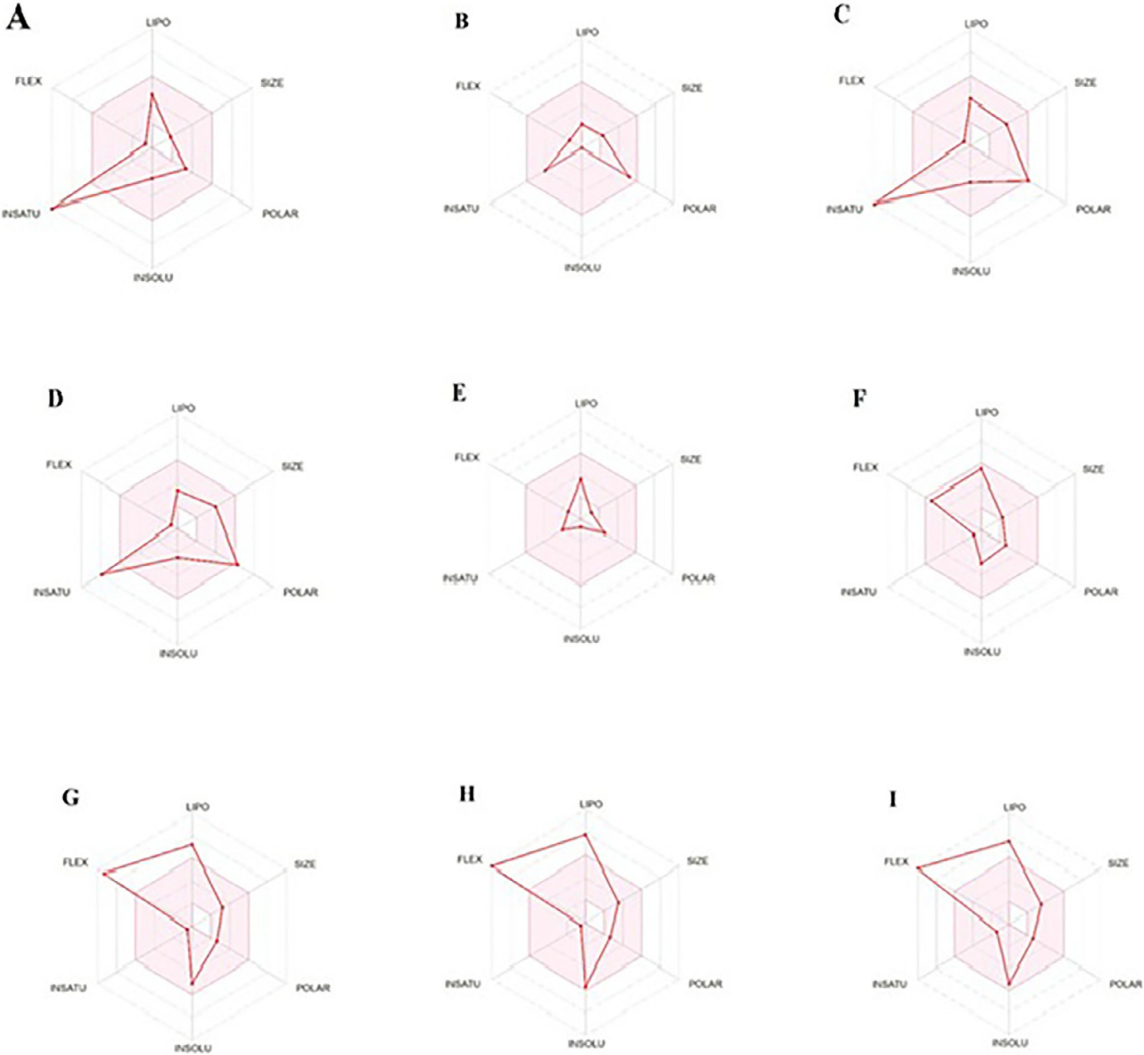
Schematic diagram of bioavailability. (A) Hydroxy benzoic acid, (B) ascorbic acid, (C) quercetin, (D) butanoic acid, (E) tri‐decanoic acid, (F) *n*‐hexadecanoic acid, (G) octadecanoic acid, (H) oleic acid, and (I) catechin.

Medicinal chemistry properties of the phytochemicals are tabulated in Table 10. Hydroxy benzoic acid exhibited 0 (PAINS) alerts; one reactive group was identified in the compound, and its synthetic accessibility was found to be 1.00, which means that it can be easily synthesized. Ascorbic acid exhibited 0 (PAINS) alerts; one reactive group was identified in the compound, and its synthetic accessibility was found to be 3.47, which means that it can be easily synthesized. Quercetin exhibited 0 (PAINS) alerts; one reactive group was identified in the compound, and its synthetic accessibility was found to be 3.23, which means that it can be easily synthesized. Catechin exhibited 0 (PAINS) alerts; one reactive group was identified in the compound, and its synthetic accessibility was found to be 3.62, which means that it can be easily synthesized. Butanoic acid exhibited 0 (PAINS) alerts; one reactive group was identified in the compound, and its synthetic accessibility was found to be 1.00, which means that it can be easily synthesized. Tri‐decanoic acid exhibited 0 (PAINS) alerts; one reactive group was identified in the compound, and its synthetic accessibility was found to be 1.67, which means that it can be easily synthesized. *n*‐Hexadecanoic acid exhibited 0 (PAINS) alerts; one reactive group was identified in the compound, and its synthetic accessibility was found to be 2.31, which means that it can be easily synthesized. Octadecanoic acid exhibited 0 (PAINS) alerts; one reactive group was identified in the compound, and its synthetic accessibility was found to be 2.54, which means that it can be easily synthesized. Oleic acid exhibited 0 (PAINS) alerts; one reactive group was identified in the compound, and its synthetic accessibility was found to be 3.07, which means that it can be easily synthesized. Figure 5 shows the radar chart of the compounds identified in HPLC and GC–MS.

**TABLE 10:** Medicinal chemistry of phytochemical determined by SwissADME database.

Medicinal chemistry				
Ligands name	Lead‐likeness	Brenk	PAINS	Synthetic accessibility
Ascorbic acid	No; 1 violation: MW<250	0 alert	0 alert	3.47
Hydroxy benzoic acid	No; 1 violation: MW<250	0 alert	0 alert	1.00
Catechin	Yes	1 alert: catechol	1 alert: catechol_A	3.62
Quercetin	Yes	1 alert: catechol	1 alert: catechol_A	3.23
Tri‐decanoic acid	No; 3 violations: MW<250, Rotors>7, XLOGP3>3.5	0 alert	0 alert	1.67
Butanoic acid	No; 1 violation: MW<250	0 alert	0 alert	1.00
Octadecanoic acid	No; 2 violations: Rotors>7, XLOGP3>3.5	0 alert	0 alert	2.54
*n*‐Hexadecanoic acid	No; 2 violations: Rotors>7, XLOGP3>3.5	0 alert	0 alert	2.31
Oleic acid	No; 2 violations: Rotors>7, XLOGP3>3.5	1 alert: isolated_alkene	0 alert	3.07

## 4 Discussions

The phytochemical analysis and in vitro investigation of the extract from the cone of *M. zotictla* providing evidence of its therapeutic efficacy in the context of diabetes and oxidative stress. Through HPLC, phenolic constituents and flavonoids were identified, and these compounds have been reported to have enzyme inhibitory and antioxidant effects. They have the ability to scavenge free radicals and modulate metabolism of carbohydrates [32]. DPPH assay was used to determine the antioxidant potential, and it was found that the extract has the ability to neutralize the oxidative stress caused by oxygen free radicals [33]. GS‐MS analysis revealed the presence of volatile components in the extracts that have been known to have antidiabetic, anti‐inflammatory, and antioxidant potential [34]. The findings of the current study suggest that *M. zotictla* is rich in phytochemical, which is attributable to the bioactivity of the plant. Furthermore, the extract showed significant inhibitory effects against *α*‐amylase enzyme comparable to standard antidiabetic agents like acarbose. This supports its potential role in controlling postprandial blood glucose levels, likely due to the interaction of phenolic compounds with digestive enzymes [35]. The antioxidant and antidiabetic efficacy of extract in the current study may be attributed to phytochemicals present in the plant, including phenolic compounds and flavonoids. Hydroxy benzoic acid, catechin, and quercetin have been reported to have potent antioxidant activity, scavenge oxygen free radicals, and protect the cell from oxidative stress [36, 37, 38]. Inhibitory effects of these compounds against *α*‐glucosidase and *α*‐amylase have been reported, and they also protect the beta‐cells of pancreas from damage [39]. Ascorbic acid has been known for its antioxidant effect associated with hyperglycemia. It has been reported that butanoic acid improves insulin sensitivity and homeostasis of glucose [40]. Oleic acid has been known for enhancing insulin signaling [41]. Collectively, these compounds act in synergy and enhance antioxidant efficacy, improve metabolism of glucose, and contribute to the antidiabetic and antioxidant capabilities of *M. zotictla*.

The SwissADME profile shows that some of the phytochemicals present in the extract did not violated Lipinski's rule of five. Drug discovery and development may strictly follow Lipinski's rule of five. Any candidate violating a single rule may decrease its permeability and will show poor absorbance [42]. This rule was developed for the evaluation of oral bio bioavailability and 90% of the orally administered drugs and many of them have passed to Phase II clinical trials [43]. SwissADME calculated essential parameters, including log *P*_o/w_, iLOGP, WLOGP, and XLOGP3, which are collectively called consensus log. The compounds were determined by HPLC and GC–MS; the log *P*_o/w_ values ranged between 1.24 and 4.34 which is indication of lipophilic tendency. Octadecanoic acid has consensus log of 5.93, which displays its highest lipophilicity. Several compounds exhibited bioavailability score of 0.85; all compounds show high gastrointestinal absorption capabilities (GI), a few compounds have the ability to cross BBB [44]. Overall, some of the compounds are in ideal range of Lipinski's rule of molecular weight, which is below 500. The second criteria are number of rotatable bonds; the range is below 10. All the compounds have shown good results, which means they are favorable for oral bioavailability, except *n*‐hexadecanoic acid, octadecanoic acid, and oleic acid, which are unfavorable for oral bioavailability. The next one is the number of hydrogen bond donor, and nd the range for oral bioavailability is below 5. All the compounds have shown good result except catechin which have six hydrogen bond donor. The next one is hydrogen bond acceptor, and the favorable range for oral bioavailability is below 10, and all the compounds showed good results and all are favorable for oral bioavailability. The next one, topological polar surface area for intestinal absorption and BBB penetration, the favorable range is below 140 Å^2^, all the compounds showed good result and all are below 140 Å^2^ [28]. Lipophilicity and water solubility are inversely proportional to each other if a compound is more lipophilic it will have less water solubility and all the compounds show moderate lipophilicity but octadecanoic acid is highly lipophilic and has low water solubility, all the compounds show good water solubility except ascorbic acid that shows high water solubility and poor lipophilicity [45]. The Pharmacokinetic studies show the compound is suitable for oral administration. A compound is considered better for oral administration which has high GI absorption and minimal interaction with efflux pumps (P‐gp) or metabolism enzyme (CYP450) to avoid drug–drug interactions. The compound which are best for oral administration are hydroxy benzoic acid, ascorbic acid, catechin, butanoic acid, and tri‐decanoic acid. The compounds that may cause some issues or interact with other drugs are quercetin, *n*‐hexadecanoic acid, octadecanoic acid, and oleic acid [28].

The current study was aimed at determination of phytochemical profiling, enzyme inhibition, antioxidant, docking studies, and SwissADME analysis. The findings of the study revealed the presence of active secondary metabolites, potential of enzyme inhibition, and radical scavenging effects. The computational studies found good interaction between the compounds identified by HPLC and GC–MS and enzyme. The SwissADME analysis further supported the medicinal chemistry of the extract.

## 5 Conclusion

In this study, *M. zotictla* cones were collected, ground, and crude extract was obtained in methanol, which was subjected to phytochemical profiling by HPLC and GC–MS analyses, followed by in vitro studies against DPPH and *α*‐amylase inhibition assays, after which molecular docking and SwissADME analyses were carried out. Phytochemical analysis shows the presence of bioactive compounds like hydroxy benzoic acid, quercetin, and ascorbic acid, which highlight the plant's therapeutic ability. The in vitro antioxidant and antidiabetic assays carried out for crude extract of *M. zotictla* had impact on inhibition of DPPH as well as *α*‐amylase and thus suggest their role in the management of oxidative stress and diabetes. The molecular docking shows interaction of the compounds with the receptors, which validates the in vitro enzyme inhibition capability. The SwissADME analysis revealed the medicinal properties of the compounds identified in the plant extract. The findings of this piece of work are limited to only in vitro and in silico studies; further in vivo studies are encouraged for better results.

## Author Contributions

**A.A. and M.Z**.: supervision of whole project and writing of resraech paper; **U.S**., **M.Z**., **A.A**., and **M.B**.: methodology; **A.K**.: supervision and interpret the total study; **H**., **M.Z**.: conceptualization; **M.Z**. and **A.K**.: software; **A.A**., **M.Z**., and **H**.: formal analysis. Authors have read and agreed to the published version of the manuscript.

## Funding

Princess Nourah bint Abdulrahman University Researchers Supporting Project (number PNURSP2026R33), Princess Nourah bint Abdulrahman University, Riyadh, Saudi Arabia.

## Conflicts of Interest

The authors declare no conflicts of interest.

## Data Availability

Data sharing not applicable to this article as no datasets were generated or analysed during the current study.
